# Anti-EGFR Antibody Efficiently and Specifically Inhibits Human TSC2^−^/^−^ Smooth Muscle Cell Proliferation. Possible Treatment Options for TSC and LAM

**DOI:** 10.1371/journal.pone.0003558

**Published:** 2008-10-29

**Authors:** Elena Lesma, Vera Grande, Silvia Ancona, Stephana Carelli, Anna Maria Di Giulio, Alfredo Gorio

**Affiliations:** 1 Laboratory of Pharmacology , Department of Medicine, Surgery and Dentistry- Polo H. San Paolo, Faculty of Medicine, University of Milan, Milan, Italy; 2 Clinical Pharmacology, IRCCS Humanitas, Milan, Italy; University of Giessen Lung Center, Germany

## Abstract

**Background:**

Tuberous sclerosis complex (TSC), a tumor syndrome caused by mutations in *TSC1* or *TSC2* genes, is characterized by the development of hamartomas. We previously isolated, from an angiomyolipoma of a TSC2 patient, a homogenous population of smooth muscle-like cells (TSC2^−/−^ ASM cells) that have a mutation in the *TSC2* gene as well as TSC2 loss of heterozygosity (LOH) and consequently, do not produce the TSC2 gene product, tuberin. TSC2^−/−^ ASM cell proliferation is EGF-dependent.

**Methods and Findings:**

Effects of EGF on proliferation of TSC2^−/−^ ASM cells and TSC2^−/−^ ASM cells transfected with *TSC2* gene were determined. In contrast to TSC2^−/−^ ASM cells, growth of *TSC2*-transfected cells was not dependent on EGF. Moreover, phosphorylation of Akt, PTEN, Erk and S6 was significantly decreased. EGF is a proliferative factor of TSC2^−/−^ ASM cells. Exposure of TSC2^−/−^ ASM cells to anti-EGFR antibodies significantly inhibited their proliferation, reverted reactivity to HMB45 antibody, a marker of TSC2^−/−^ cell phenotype, and inhibited constitutive phosphorylation of S6 and ERK. Exposure of TSC2^−/−^ ASM cells to rapamycin reduced the proliferation rate, but only when added at plating time. Although rapamycin efficiently inhibited S6 phosphorylation, it was less efficient than anti-EGFR antibody in reverting HMB45 reactivity and blocking ERK phosphorylation. In TSC2^−/−^ ASM cells specific PI3K inhibitors (e.g. LY294002, wortmannin) and Akt1 siRNA had little effect on S6 and ERK phosphorylation. Following *TSC2*-gene transfection, Akt inhibitor sensitivity was observed.

**Conclusion:**

Our results show that an EGF independent pathway is more important than that involving IGF-I for growth and survival of TSC^−/−^ ASM cells, and such EGF-dependency is the result of the lack of tuberin.

## Introduction

Tuberous sclerosis complex (TSC) is an autosomal dominant disorder characterized by the development of hamartomas, which are unusual tumor-like growths found in a variety of tissues [1]. The two genes implicated in tuberous sclerosis, *TSC1* and *TSC2*, with loci on chromosomes 9q34 (TSC1) and 16p13.3 (TSC2) respectively [2], [3], participate in the control of cell size via the insulin/p70 ribosomal S6 kinase 1 (S6K1) pathway [4]. The *TSC1* gene codes for hamartin, a 130-kDa hydrophilic protein with no homology to tuberin, the 200-kDa protein encoded by *TSC2* gene [1]. Tuberin and hamartin function together as a heterodimer to inhibit mammalian target of rapamycin (mTOR)-mediated signaling to S6K [5], [6]. This complex acts downstream of PI3K and Akt, and upstream of Rheb, mTOR and p70S6K1. In mammalian cells, Rheb overexpression greatly enhances mTOR signaling. The lack of tuberin or hamartin promotes p70S6K activation and S6 phosphorylation, and increased DNA synthesis in cultures of patient [7], and established cell lines [8].

Insulin and other growth factors are thought to regulate the phosphorylation of S6K1 and 4E-binding protein 1 (4EBP1) through the PI3K-signaling pathway via phosphorylation and activation of Akt [9], [10]. Tuberin regulates and is, itself, regulated by p42/44 mitogen-activated protein kinase (MAPK). Activation of the MAPK pathway by growth factors leads to phosphorylation of two MAPKs, Erk-1 ^(p44mapk)^ and Erk-2 ^(p42mapk)^, which translocate to the nucleus to regulate gene transcription. The tuberin-dependent phosphorylation of B-raf and p42/44 MAPK, the p42/44 MAPK-dependent direct phosphorylation of tuberin and that mediated through S6K suggest an interaction between MAPK pathway and tuberin [11], [12], [13]. Ras/MAPK and PI3K pathways converge on the tumor suppressor tuberin to inhibit its function [12]. MAPK-dependent phosphorylation of tuberin may lead to somatic inactivation of the hamartin/tuberin complex in tuberous sclerosis complex-associated brain hamartomas that have activated MEK1 and ERK1 [14].

We have isolated and characterized a homogenous population of human smooth muscle like-cells (TSC2^−/−^ASM cells) from an angiomyolipoma obtained from a TSC2 patient after total nephrectomy. The cells bear a germline TSC2 mutation, consisting of a single base-pair change resulting in replacement of lysine 698 with a stop codon (K698X), as well as loss of heterozygosity (LOH), and do not express tuberin [15]. These cells present the typical constitutive activation of S6K1 and S6, and greater phosphorylation of Akt and ERK, contain melanocyte antigens and react with monoclonal antibody HMB45, which recognizes the gp100 protein. When grown in culture, these cells appear not to undergo senescence based on morphological, biochemical, and pharmacological data. TSC2^−/−^ASM cells require epidermal growth factor (EGF) in the growth medium for proliferation, and its replacement with IGF-I greatly reduces cell growth. IGF-I, however, is important for these cells. They secrete IGF-I, which may act as a survival factor by promoting the expression of survivin [16]. Blockade of either EGF receptors or IGF-I receptors with specific antibodies resulted in total cell death within 12 days [15].

In the present study, we aimed at evaluating the role of EGF pathway in growth and survival of TSC2^−/−^ ASM cells, and the relationship between the lack of tuberin and the dependency on EGF by these cells. In addition we aimed at understanding the role of PI3K pathway. Here, we show that the EGF requirement for human TSC2^−/−^ASM cell growth is caused by lack of tuberin. Blockade of the EGF receptor inhibited cell proliferation and S6 and ERK phosphorylation, and caused a rapid reversion of phenotype, as determined by HMB45 reactivity. Rapamycin affected cell growth only when applied at plating time. Through the inhibition of PI3K with specific inhibitors or transient transfection of the cells with siRNA oligomer for Akt1, we show that proliferation of human TSC2^−/−^ASM cell is not dependent upon the functionality of PI3K pathway. In contrast, ERK plays a key role in the regulation of growth. In TSC2^−/−^ ASM cells the PI3K inhibition, by PI3K inhibitors and siRNA Akt1, was inefficient, while its functionality was re-stablished following transformation with the *TSC2* gene.

## Materials and Methods

### Cell lines

TSC2^−/−^ASM cells were isolated, characterized and grown, as previously described [15]. They were obtained from a renal angiomyolipoma during total nephrectomy from a 42-year-old female with a history of TSC2 who had given written informed consent according to the Declaration of Helsinki. The study was approved by the Institutional Review Board of Milan's San Paolo Hospital. The culture medium contained a 50/50 mixture of DMEM/Ham F12 (Euroclone; Paignton; United Kingdom) supplemented with hydrocortisone (2×10^−7^ M) (Sigma-Aldrich, St. Louis, USA), epidermal growth factor (10 ng/ml) (Sigma-Aldrich), sodium selenite (5×10^−8^ M) (Sigma-Aldrich), insulin (25 µg/ml) (Sigma-Aldrich), transferrin (10 µg/ml) (Sigma-Aldrich), ferrous sulfate (1.6×10^−6^ M) (Sigma-Aldrich), and 15% fetal bovine serum (Euroclone) as described by Arbiser et al. [17]. The CT/G human aorta vascular smooth muscle cells (VSMCs) (ATCC, Manassas, USA) were grown with F12 medium containing 10% fetal bovine serum, 100 U/ml penicillin and streptomycin and 2 mM L-glutamine (Euroclone). GP2-293 packaging cells (BD-Biosciences-Clontech, Palo Alto, CA USA) were grown in low-glucose DMEM medium with 10% fetal bovine serum, 100 U/ml each penicillin and streptomycin and 2 mM L-glutamine.

### Generation of tuberin-expressing cell lines

Transfection was performed as previously described by Astrinidis et al. (2002) [18]. Briefly, GP2-293 cells were co-transfected with 2 µg of the retroviral vector pMSCVneo (a gift from Dr. A. Astrinidis and E.P. Henske, Fox Chase Cancer Center, Philadelphia, PA, USA), 1 µg pVSV-G (BD-Biosciences-Clontech) encoding the viral glycoprotein, and 6 µl FuGene6 (Roche, Indianapolis, IN, USA). Replication-deficient retroviruses were collected from the culture after 72 h and applied to subconfluent TSC2^−/−^ASM cells in the presence of 8 µg/ml polybrene (Sigma-Aldrich). Cells were transduced with empty pMSCVneo vector as a control, or a pMSCVneo construct containing the coding region of the human *TSC2* gene. Stable clones were selected for 2 weeks in the presence of 100 µg/ml G418 (Sigma).

### Evaluation of Cell Proliferation

Cellular proliferation was evaluated by counting at least 400–500 cells in a Neubauer chamber. TSC2^−/−^ ASM and transfected-cells were grown with or without EGF (10 ng/ml) or substituting IGF-1 (50 ng/ml) to EGF in the culture medium for 30 days. Cells were counted after 7, 15, 20 and 30 days. Each data point was the mean of three independent experiments.

The actions of rapamycin (Rapamune-Sirulimus, Wyeth Europa, UK; 1 ng/ml) and anti-EGFR (clone 225; 5 µg/ml; Calbiochem, Darmstadt, Germany) were evaluated by adding the drugs to TSC2^−/−^ ASM cells at plating time or 3 hours after plating for 12 days. The effect of different doses of rapamycin (5, 10, and 20 ng/ml) was evaluated by adding rapamycin 3 hours after plating for 12 days. Cells were counted after 2, 5, 7, 10, 12 days. Each data point was the mean of three independent experiments.

### Immunofluorescence Microscopy

Cells were cultured on glass slides, then permeabilized with 70% methanol for 10 min, and dried in air. The primary antibodies against α-actin (1∶100; Sigma), HMB45 (1∶100; Dako, Carpinteria, CA USA), and tuberin (C-20) (1∶100; Santa Cruz, Santa Cruz, CA, USA) were applied overnight at 4°C. The samples were incubated for three hours at room temperature with fluorescein isothiocyanate (FITC)-conjugated rabbit anti-mouse antibody (Chemicon, Temecula, CA, USA) for HMB45, and α-actin and rhodamine-conjugated goat anti-rabbit antibody (Chemicon) for studies with tuberin. After washing, the slides were mounted in FluorSave reagent (Calbiochem, Darmstadt, Germany).

HMB45 reactivity was evaluated by immunofluorescence. TSC2^−/−^ ASM cells were incubated for 48 hours or 5 days. Rapamycin (1 ng/ml) or anti-EGFR (5 µg/ml) was added at plating time or 3 hours after plating. HMB45 labeling was defined as strong (++), intermediate (+) and negative (−). HMB45 reactivity was evaluated also after TSC2-transfection. Quantification of HMB45 intensity was achieved with a confocal microscope (Leica TCS-SP2) and analyzed by Leica Confocal software using the profile through stack/series methods as follows: 30 equal polygonal areas covering the cells were randomly selected for each image. The fluorescence intensity values of the selected cells were quantified and the mean and the standard deviation of the data were calculated by the GraphPad software. The level of statistical significance was determined by Student's *t*-test.

### Cell treatment

For evaluating the effects of anti-EGFR and rapamycin on TSC2^−/−^ ASM cells rapamycin (1 ng/ml) or anti-EGFR antibody (5 µg/ml) were added to the culture medium at plating time or 3 hours after plating for 24 or 48 hours.

For PI3K pathway evaluation , TSC2^−/−^ ASM and transfected cells were incubated for 2 hours with IGF-I (50 ng/ml) with or without LY294002 (20 µM), PD98059 (30 µM), or wortmannin (80 nm or 320 nM). The experiments were performed in complete medium or after 24 hours starvation.

### Western blotting

Cells were lysed in lysis buffer (5 mM EDTA, 100 mM deoxycholic acid, 3% SDS). Samples (50 µg per lane) were boiled for 5 min, and analyzed by 10% SDS-PAGE. After transfer to nitrocellulose membranes (Amersham, Arlington Height, USA) and blocking at room temperature for 3 h with 5% dry milk (Merck, Darmstadt, Germany), membranes were incubated overnight at 4°C with antibodies against tuberin (C-20) (1∶100; Santa Cruz), phospho-Akt (1∶1000; Cell Signaling; Beverly, CA, USA), Akt (1∶200; Santa Cruz), phospho-S6K (1∶1000; Cell Signaling), S6K1 (1∶1000; Cell Signaling), phospho-S6 (1∶1000; Cell Signaling), S6 (1∶1000; Cell Signaling), phospho-extracellular signal-regulated kinase 1 and 2 (ERK1/2) (1∶1000; Cell Signaling), EGF receptor (Cell Signaling), IGF-I receptor (1∶1000; Cell Signaling), phospho-PTEN (Cell Signaling), PTEN (1∶1000; Cell Signaling) or β-actin (Cell Signaling). Membranes were washed, then incubated for 1 hour with the appropriate secondary antibodies (1∶10000; Chemicon). The reaction was quantified using the SuperSignal West Pico Chemiluminescent Substrate (Pierce, Rockford, IL, USA). Densitometric analysis was performed by Kodak MJ project program. Data were expressed as optical density.

### siRNA transfection

TSC2^−/−^ASM or *TSC2*-transformed cells were transfected with small interfering RNA (siRNA) to reduce Akt isoforms. Custom-made validated siRNAs for Akt1 isoforms were obtained from Ambion (Austin, USA). The siRNA duplexes (50 nM) were introduced with Lipofectamine 2000 (Invitrogen Corporation, Carlsbad, USA) per manufacturer's protocol. The cells were assayed after 72 hours.

### Statistical Analysis

All data are expressed as means values±SEM, and were statistically analyzed using Student's *t*-test; significance is indicated for *P* values of *<0.005, **<0.01 and ***<0.001.

## Results

### Development of cells with stable expression of tuberin from human TSC2^−/−^ASM cells

**Figure 1:**
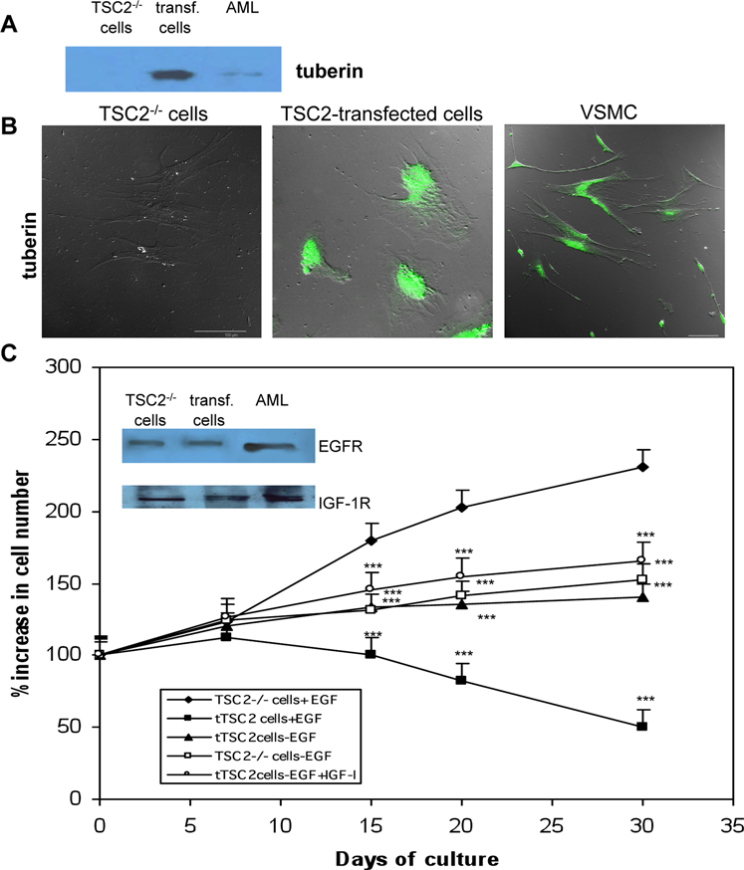
Development of smooth muscle cells with stable human tuberin expression from human TSC2^−/−^ASM cells. A: Expression of tuberin was evaluated by Western blotting in TSC2^−/−^ASM cells, *TSC2*-transfected cells (transf. cells), and AML. The slight expression in AML is due to the presence of non-LOH TSC2 cells. B: Tuberin was undetectable by immunocytochemistry in TSC2^−/−^ASM cells, but it was expressed in these cells following TSC2 transfection. Aorta smooth muscle cells (VSMC) show immunolabeling of tuberin. C: Evaluation of EGF effects following *TSC2*-gene transfection in TSC2^−/−^ASM cells. Insert: EGFR and IGF-IR are expressed in TSC2^−/−^ASM cells, *TSC2*-transfected cells, and AML. TSC2^−/−^ASM cells require, and *TSC2*-transfected cells (tTSC2 cells) do not require, EGF in the medium for cell proliferation. IGF-I promotes proliferation of TSC2-transfected cells and normal smooth muscle cells. EGF has a negative effect on proliferation of transfected cells as it does on normal human smooth muscle cells. Experiments were done three times each. Mean values±SEM. Significant differences (***P<0.001) versus control were evaluated by student's *t*-test.

TSC2^−/−^ ASM cell proliferation required EGF in the culture medium [15]. To study the correlation between the proliferative role of EGF and tuberin in ASM cells, the full-length wild-type human tuberin was successfully expressed in TSC2^−/−^ASM cells following retroviral transduction of the *TSC2* gene (Fig. 1A and 1B). Expression and cellular distribution of tuberin was comparable to that of normal human vascular smooth muscle cells (VSMCs) and has been observed for up to 4 months. It was localized primarily in the cell body and to a smaller extent in the peripheral processes (Fig. 1B). The expression of EGF- and IGF-I-receptors in TSC2^−/−^ASM cells was not affected by *TSC2* transfection (Fig. 1C); however, the responses to these factors were significantly different in transfected and non-transfected cells. Addition of EGF to the growth medium promotes proliferation of human TSC2^−/−^ASM cells [15], but, following *TSC2* transfection, EGF inhibited proliferation (Fig. 1C). This action of EGF was reported previously for VSMCs [15] and apparently results from EGF-dependent early DNA synthesis, accompanied by minimal cell division for 0 to 4 days, that leads to subsequent cell cycle arrest [19]. TSC2^−/−^ASM and *TSC2*-transfected cells grew at a comparable rate in the medium lacking EGF. The addition of IGF-I to the growth medium slightly improved the proliferation rate of *TSC2*-transfected cells (Fig. 1C), as reported previously for normal human smooth muscle cells [15].

**Figure 2:**
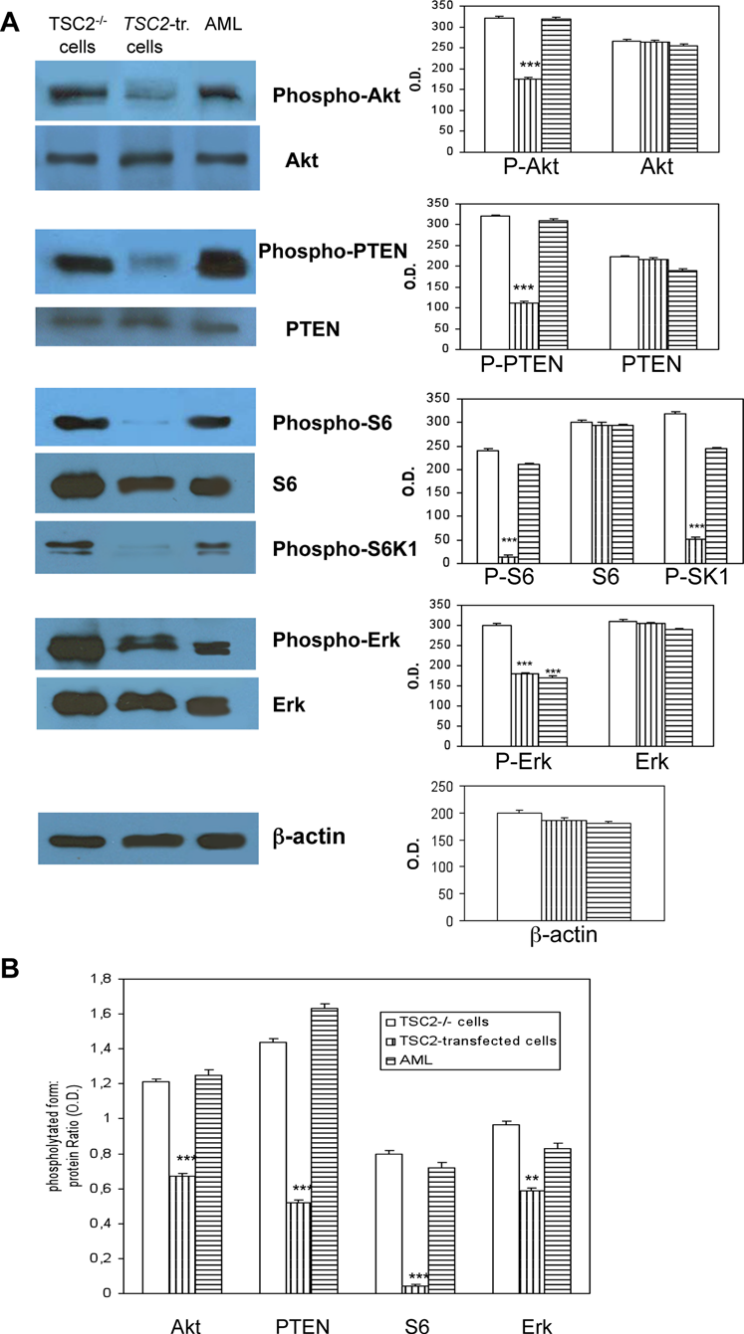
Biochemical rearrangements promoted by *TSC2* transfection. A: Constitutive phosphorylation of Akt, PTEN, S6K1, and S6 was markedly decreased by TSC transfection; interestingly also the extent of ERK phosphorylation was reduced. B: Also the phosphorylated form to total protein ratio shows a marked reduction of the extent of phosphorylation caused by *TSC2*-gene transfection. The blots are representative of three separate experiments.

Enhanced phosphorylations of Akt, S6K1 and S6 can be considered a biochemical marker of TSC2 deficiency [5], [7], [15], [20], [21]. The extent of Akt, S6K1 and S6 phosphorylation was reduced in transformed cells compared to TSC2^−/−^ASM cells, while the expression of Akt and S6 proteins was unaltered (Fig. 2A). PTEN phosphorylation was markedly lower in *TSC2*-transfected cells compared to TSC2^−/−^ ASM cells, and this was similar to what was observed with Akt. As expected, the extent of ERK phosphorylation was reduced by TSC2-transfection (Fig. 2A). Thus, reduced Akt, PTEN, ERK, and S6 phosphorylation was associated with *TSC2* transfection as evidenced by the reduced ratios of phosphorylated to specific protein level (Fig. 2B).

### Effect of anti-EGFR antibody and rapamycin on TSC2^−/−^ASM cell growth and phenotype

**Figure 3:**
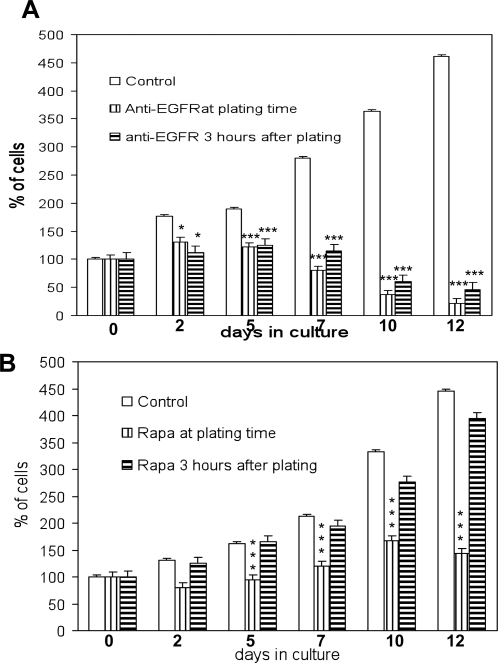
Effect of anti-EGFR antibody and rapamycin (rapa) on TSC2^−/−^ASM cell growth. A: 72 hour incubation: anti-EGFR antibody incubation (5 µg/ml) at plating time and 3 hours after plating inhibited proliferation and caused a marked TSC2^−/−^ASM cell death. B: Incubation at plating time with rapamycin (1 ng/ml) slowed significantly TSC2^−/−^ASM cell growth, while its addition at 3 hours after plating failed to affect proliferation. Mean values±SEM. Significant differences (*P<0.05, ***P<0.001) versus control were evaluated by student's *t*-test.

From observations reported above, it appears that EGFR is involved in proliferation of human TSC2^−^/^−^ ASM cells. Inhibitory effects of anti-EGFR antibody and rapamycin on proliferation were observed by adding them to the culture medium prior or after cell attachment to the dish. We chose the different timing of application because cells may change the sensitivity to drugs following adhesion. The addition of anti-EGFR antibody (5 µg/ml) either at plating time or 3 hours after plating, led to an early inhibition of TSC2^−/−^ASM cell proliferation, followed by almost complete cell death within 12 days (Fig. 3A). In contrast, the addition of rapamycin (1 ng/ml) (at plating time), which inhibits mTOR, slowed the proliferation rate of TSC2^−/−^ASM cells, while a delay of 3 hours eliminated the effects of rapamycin on proliferation (Fig. 3B). The 1 ng/ml concentration of rapamycin was used because it was able to reduce the TSC2^−/−^ ASM cell growth rate to levels similar to controls in presence or absence of EGF in the growth media as shown in Lesma et al. [15]. We have also employed higher concentrations (up to 20 ng/ml) of rapamycin, and only the highest concentrations of 10 and 20 ng/ml were able to slightly reduce the proliferation ability of TSC2^−/−^ ASM cells (Figure S1). All these concentrations, similarly, to 1 ng/ml were capable of blocking the constitutive phosphorylation of S6 (data not shown).

**Figure 4:**
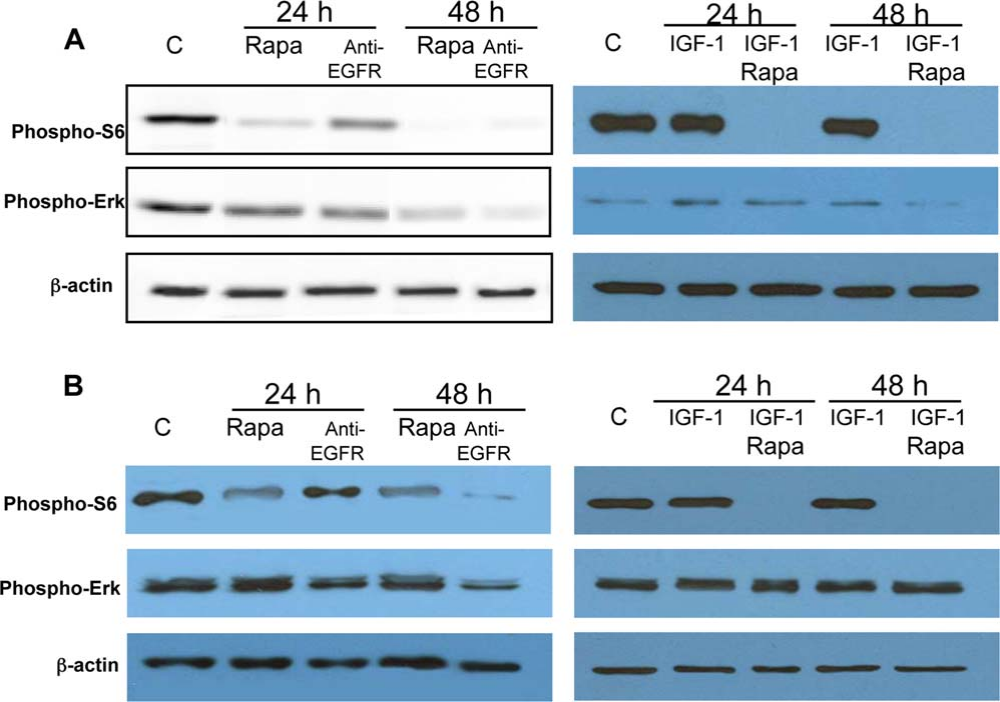
Effect of rapamycin (rapa) (1 ng/ml) and anti-EGFR (5 µg/ml) and IGF-I (50 ng/ml) on phosphorylation of S6 and ERK. The blots are representative of three experiments. A: Incubation with rapamycin at plating time for 24 or 48 hours inhibited S6 phosphorylation even in the presence of IGF-I. Also anti-EGFR antibody affected the extent of S6 phosphorylation at 48 hours. ERK phosphorylation is reduced by both agents after 48 hours. B: When the drugs are added 3 hours after cell plating, rapamycin reduces S6 phosphorylation in 24 and 48 hours incubation but failed to affect ERK phosphorylation. Anti-EGFR antibody inhibited both S6 and ERK phosphorylation within 48 hours. IGF-I alone did not affect the extent of S6 and ERK phosphorylation.

Exposure, at plating time, to rapamycin (1 ng/ml) and anti-EGFR antibody greatly reduced S6 and ERK constitutive phosphorylation at 48 hours (Fig. 4A). A delay of 3 hours in drug addition however, revealed different responses of the two reagents. Anti-EGFR antibody reduced both S6 and ERK phosphorylation in 48 hours, while rapamycin reduced S6 phosphorylation but no longer affected ERK activation (Fig. 4B). S6 phosphorylation inTSC2^−/−^ ASM cells was not affected by IGF-I (Figure 4A), while ERK phosphorylation was slightly increased (Fig. 4A).

**Figure 5:**
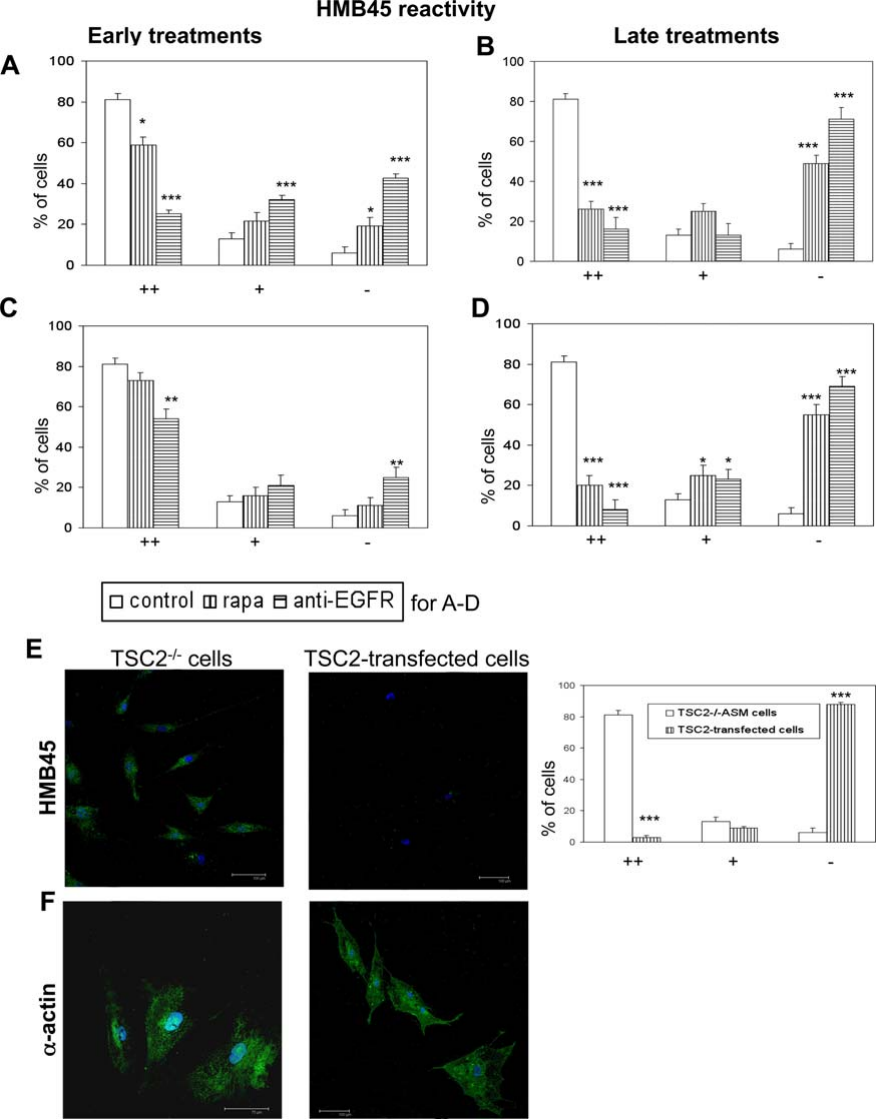
Effect of rapamycin (1 ng/ml) and anti-EGFR antibody (5 µg/ml) on reactivity with monoclonal HMB45 in human TSC2^−/−^ASM cells. Positive cells are indicated as ++, slightly positive as + and negative as −. A: Incubation at cell plating time with anti-EGFR for 48 hours decreases markedly the number of cells reacting to HMB45 while the number of negative cells is strongly increased. Rapamycin is markedly less than anti-EGFR antibody. B: Incubation for 5 days with either rapamycin or anti-EGFR antibody, applied at cell plating time, caused a significant reduction of HMB45 reactivity in both cases. C: 48 hours exposure to rapamycin or anti-EGFR antibody, both applied 3 hours after plating, caused only a slight reduction of HMB45 reactivity of TSC2^−/−^ASM cells. D: Exposure to rapamycin or anti-EGFR antibody applied 3 hours after cell plating for 5 days caused a drastic reduction in HMB45 reactivity of TSC2^−/−^ASM cells. E: Transfection of TSC2^−/−^ ASM cells with *TSC2* gene significantly reduced HMB45 reactivity. F: α-actin was present in both TSC2^−/−^ASM and *TSC2*-transfected cells. Mean values±SEM. Significant differences (*P<0.05, **P<0.01, ***P<0.001) versus control were evaluated by student's *t*-test.

To correlate the effects of rapamycin and anti-EGFR antibody on proliferation to changes in TSC2^−/−^ASM cell phenotype, gp100 expression, quantified with HMB45 antibody, was examined after 48 hours and 5 days of drug exposure. TSC2^−/−^ASM cells are reactive with HMB45 antibody, a marker of TSC and LAM cells [15], [22]. HMB45 reactivity was quantified as strong (++), intermediate (+) and negative (−). About 80% of untreated TSC2^−/−^ASM cells were strongly reactive with HMB45, 13% slightly labelled, and 6% were negative (Fig. 5A). In contrast, after a 48 hour incubation with anti-EGFR antibody added at plating time, 25% of the cells were positive, 32% had an intermediate labelling and 43% were negative (Fig. 5A). A 48 hour incubation with rapamycin, added at plating time, reduced HMB45-reactive cells to 59%, while 19% were negative and 22% were slightly labelled (Fig. 5A). After 5 days of exposure to rapamycin or anti-EGFR antibody added at plating time, 49% and 71%, of TSC2^−/−^ ASM cells, respectively, were not reactive with HMB45; anti-EGFR antibody was still more effective (Fig. 5B). When anti-EGFR antibody or rapamycin was added to the medium 3 hours after plating the reversion of HMB45 reactivity was less marked at earlier times (Fig. 5C), but after a 5 day incubation, both agents suppressed reactivity (Fig. 5D). Following transformation with *TSC2* gene, the percentage of TSC2^−/−^ ASM cells negative for the immunoblotting of gp100 protein was greater than 80%, while actin expression was unaffected (Fig. 5E and F). The observed differential sensitivity to the effects of delayed addition of rapamycin on proliferation and gp100 protein expression in TSC2^−/−^ASM cells suggests that these molecular phenotypes may be regulated differently by TORC and EGFR.

### Role of PI3K pathway in TSC2^−^/^−^ ASM cells

**Figure 6:**
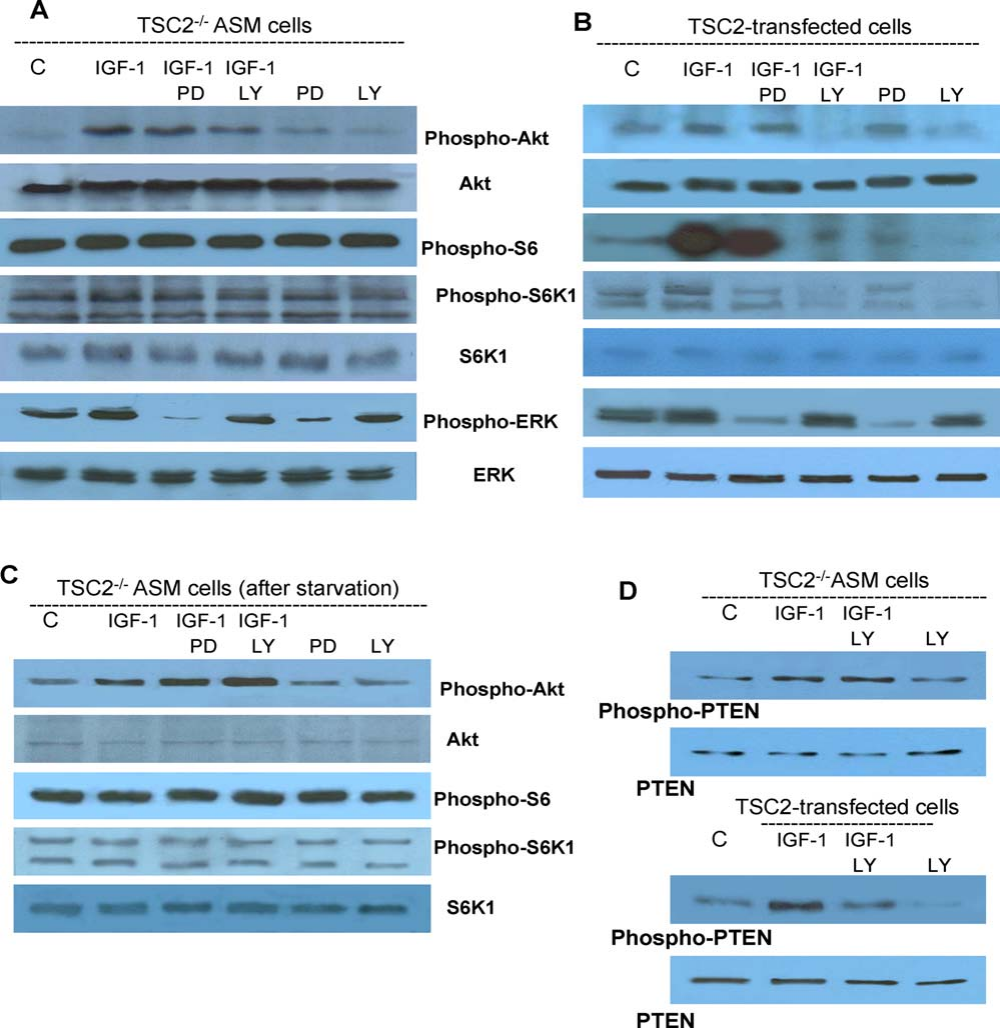
Phosphorylation of Akt, SK1 and S6 in TSC2^−/−^ASM cells and following *TSC2* transfection. A: Incubation of TSC2^−/−^ASM cells for 2 hours with IGF-I (50 ng/ml) induced Akt phosphorylation but did not affect S6K1 and S6 phosphorylation. Incubation with PI3K inhibitor, LY294002 (LY; 20 µM), or ERK inhibitor, PD98059 (PD; 30 µM), did not influence the activation of Akt, S6K1 and S6. Expression of Akt, and S6K1 was not altered by any treatment. B: Following *TSC2* transfection, LY290042 antagonized IGF-I-mediated and basal phosphorylation of Akt, S6K1, and S6. Expression of Akt, and S6K1 proteins were unaltered. C: Following 24 hours of starvation, phosphorylation of S6K1 and S6, and expression of Akt and S6K1 proteins were unaltered by 2 hour incubation with IGF-I in presence or absence of LY290042 or PD98059. Increased phosphorylation of Akt caused by IGF-I, was unaffected by incubation with LY290042 or PD98059. D: PTEN regulation in TSC2^−/−^ASM cells. In TSC2^−/−^ASM cells, phosphorylation of PTEN was increased by incubation for 2 hours with IGF-I (50 ng/ml), and this was not inhibited by LY290042 (20 µM) as observed above for Akt phosphorylation. Following *TSC2* transfection, LY290042 effectively inhibited basal and IGF-I promoted phosphorylation of PTEN as described above for Akt.

Binding of IGF-I to its receptor stimulates the intrinsic tyrosine kinase activity of the receptor, leading to phosphorylation of several substrates that activate downstream intracellular signaling through the PI-3K or Grb2-SOS, ERK1/2 pathways [23]. Incubation of TSC2^−/−^ ASM cells with IGF-I (50 ng/ml) for 2 hours, led to activation of PI3-K, and phosphorylation of Akt on Ser473 (Fig. 6A). LY294002, a cell-permeable PI-3K inhibitor, at 20 µM, slightly inhibited IGF-I-mediated Akt activation without affecting basal Akt phosphorylation (Fig. 6A and C). The lack of effects of LY294002 and PD98059 on Akt phosphorylation was confirmed by the densitometric analysis from 3 experiments (Figure S2). Higher concentrations of LY294002 (100 µM) were no more effective than 20 µM (data not shown). The extent of S6 and S6K1 phosphorylation was unaffected by treatment with IGF-I, and LY294002 or PD98059, a specific ERK inhibitor (Fig. 6A and C). The PI-3K inhibitor, wortmannin, a fungal metabolite, which irreversibly binds mammalian PI-3K and inhibits phosphorylation of several other substrates (e.g. PLA_2_, PLD, myosin chain kinase, plekstrin) [24], [25] at 80 nM and 320 nM, in presence or absence of IGF-I, did not affect Akt phosphorylation or expression (data not shown).

In contrast to these findings, IGF-I-promoted Akt phosphorylation was fully LY294002-sensitive in *TSC2*-transformed cells (Fig. 6B). LY294002 (20 µM) reduced both basal and IGF-I-induced phosphorylation of Akt, S6 and S6K1 (Fig. 6B). Similar to the findings with Akt, the basal and IGF-I-induced increase of S6 and S6K1 phosphorylation was also markedly reduced by LY294002 addition to transfected cells (Fig. 6B).

To evaluate the role of ERK1/2, TSC2^−/−^ ASM cells were incubated with IGF-I and PD98059, which blocks MEK1 activity and thus ERK phosphorylation and activation [26]. When 30 µM or 90 µM PD98059 was added for 2 hours to the culture medium in the presence or absence of IGF-I, no effect was observed on phosphorylation of Akt, S6 and S6K1 (Fig. 6A, data not shown). In *TSC2*-transfected cells, PD98059 did not affect Akt phosphorylation, but IGF-I-induced phosphorylation of S6 and S6K1 was blocked. Expression of Akt and S6K1 was not altered by any treatment (Fig. 6A, B). Following serum deprivation for 24 hours, LY294002 or PD98059 failed to affect Akt, S6K and S6 phosphorylation in IGF-I-treated TSC2^−/−^ ASM cells (Fig. 6C). In TSC2^−/−^ and *TSC2*-transfected ASM cells ERK phosphorylation was slightly increased by IGF-I exposure and inhibited by PD98059 (Fig. 6A and B).

PTEN phosphatase acts as a negative regulator of the PI-3K signaling pathway by dephosphorylating the second messengers phosphatidylinositol-3,4,5-trisphosphate [PtdIns(3,4,5)P_3_] and phosphatidylinositol-3,4-bisphosphate [PtdIns(3,4)P_2_], thereby opposing PI-3K function [27]. It has been proposed that greater Akt phosphorylation in TSC2^−^/^−^ cells might be related to reduced PTEN activity [28]. A 2 hour exposure to IGF-I (50 ng/ml) enhanced PTEN phosphorylation in TSC2^−/−^ASM cells in an LY294002-insensitive manner (Fig. 6D). Following transformation of cells with *TSC2*, LY294002 effectively blocked basal and IGF-I-promoted phosphorylation of PTEN (Fig. 6D). These data suggest that the extent of PTEN phosphorylation changes in parallel with Akt activity. Higher levels of Akt phosphorylation were associated with greater PTEN phosphorylation and vice versa.

**Figure 7:**
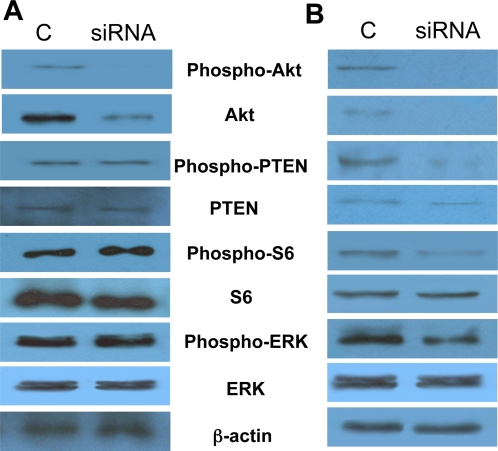
Transient transfection with siRNA for Akt1 in TSC2^−/−^ASM and *TSC2*-transfected cells. A: In TSC2^−/−^ASM cells siRNA for Akt1 drastically inhibited Akt expression and phosphorylation, but it did not affect PTEN, S6 and ERK expression and activation. B: In *TSC2*-transfected cells siRNA for Akt1 inhibited the level and the phosphorylation of Akt and S6 phosphorylation.

To study further the role of AKT in TSC2^−^/^−^ ASM cells, endogenous Akt1 gene expression was inhibited by transient transfection with Akt siRNA in the presence of EGF (Fig. 7). Akt1 siRNA reduced both Akt protein expression and phosphorylation, indicating a greater preponderance of Akt1 in TSC2^−/−^ASM cells. Phosphorylation and expression of S6 and ERK were not affected by Akt1 siRNA (Fig. 7A). The siRNA in *TSC2* transfected cells caused the drop of both Akt and phospho-Akt levels that, differently that in TSC^−/−^ ASM cells, is accompanied by the reduction in the extent of S6 phosphorylation (Fig. 7B). Such effect was also observed in normal vascular smooth muscle cells VSMCs (data not shown).

## Discussion

We had previously reported the isolation of TSC2^−/−^ASM cells from an angiomyolipoma of a TSC patient [15]. These cells have been grown for the past 4 years, and the original morphological, genetic and biochemical characteristics, such as reactivity to anti-α-actin and HMB45 antibodies, and EGF-dependent growth, were fully maintained.

Deregulation of the pathways that control cell proliferation and survival, such as those regulated by Ras/MAPK and PI3K, is a major characteristic of abnormal tumor growth and following mutations of tumor-suppressor genes leads to the formation of benign, rather than malignant, tumors. The rate of proliferation of TSC2^−^/^−^ ASM cells is sensitive to the addition of EGF to the growth medium, while secretion of IGF-I is involved in survival [15], [16]. Here, we report that the viral-induced expression of tuberin in TSC2^−/−^ ASM cells greatly reduced the growth rate, EGF ceased to be a required growth factor, and instead, it inhibited proliferation. These results support the original hypothesis that the EGF requirement for TSC2^−/−^ASM cell proliferation is dependent upon the lack of tuberin. Similar to what it is observed in normal smooth muscle cells, IGF-I slightly enhanced the proliferation rate of transfected cells [15], [29]. *TSC2*-transfection reversed the typical features of human TSC2^−/−^ASM cells, i.e. the abnormal EGF-dependent proliferation, constitutive S6 phosphorylation, and reactivity with HMB45 without affecting the smooth muscle phenotype.

Further extending the previous study [15], we evaluated the action of anti-EGFR antibody and rapamycin in regulating TSC2^−/−^ ASM cell growth. EGFR is an important target of anticancer therapy. The EGFR signalling pathways regulate cell differentiation, proliferation, migration, angiogenesis and apoptosis, all of which become deregulated in cancer cells [30]. One current strategy to block cancer growth involves the use of antibodies against the extracellular domain of EGFR, which compete with ligand for receptor binding, thereby preventing kinase activation [30]. As an example, the antibody used in this study, monoclonal antibody C225, has a high affinity for EGFR and has been shown to be efficacious in several types of cancer, particularly colorectal and head and neck cancers [30]. Rapamycin specifically inhibits mTOR, while anti-EGFR blocks a complex pathway that involves ERK, RSK1 and tuberin [31]. Rapamycin has been shown to induce apoptosis, decrease proliferation, and reduce tumor size in the Eker rat model and in *TSC2* ^+/−^ mice [32], [33]. Since mTOR is directly activated upon loss of hamartin or tuberin, rapamycin has been identified as a potential therapeutic agent for TSC and LAM. It has been recently reported that angiomyolipomas of TSC patients regressed partially during rapamycin treatment, but the volume increased again after the therapy was stopped [34]. Our data indicate that anti-EGFR antibody has a greater ability than rapamycin to inhibit proliferation of TSC2^−^/^−^ ASM cells, counteract the phosphorylation of S6 and ERK, and reduce HMB45 reactivity. Furthermore, the anti-EGFR antibody-dependent reduction of the proliferation rate led to the progressive death of human TSC2^−/−^ASM cell when added to the medium either at plating time or 3 hours after plating. In contrast, rapamycin reduced significantly cell proliferation only when added prior cell attachment. The delayed application of rapamycin did not affect TSC2^−/−^ ASM cell proliferation although S6 constitutive phosphorylation is inhibited. This may be explained by the activity of MAPK in these cells. Anti-EGFR antibody and rapamycin, however, were clearly less efficient than *TSC2*-transfection.

Consistent with the concept that S6K1 activation is PI3K independent in tuberin-deficient cells, Akt1 siRNA did not interfere with S6 phosphorylation, while PTEN phosphorylation in TSC2^−/−^ and TSC2-transfected cells followed the activation pattern of Akt. Exposure to IGF-I enhanced Akt phosphorylation of both TSC2^−/−^ ASM cells and those virally transfected with TSC2 gene; however, PI3K inhibitors, such as LY294002 and wortmannin, were able to affect this process only in TSC2-transfected cells. Thus, it appears that viral-induced expression of tuberin in TSC2^−^/^−^ ASM cells modifies cellular function and sensitivity to drugs.

**Figure 8:**
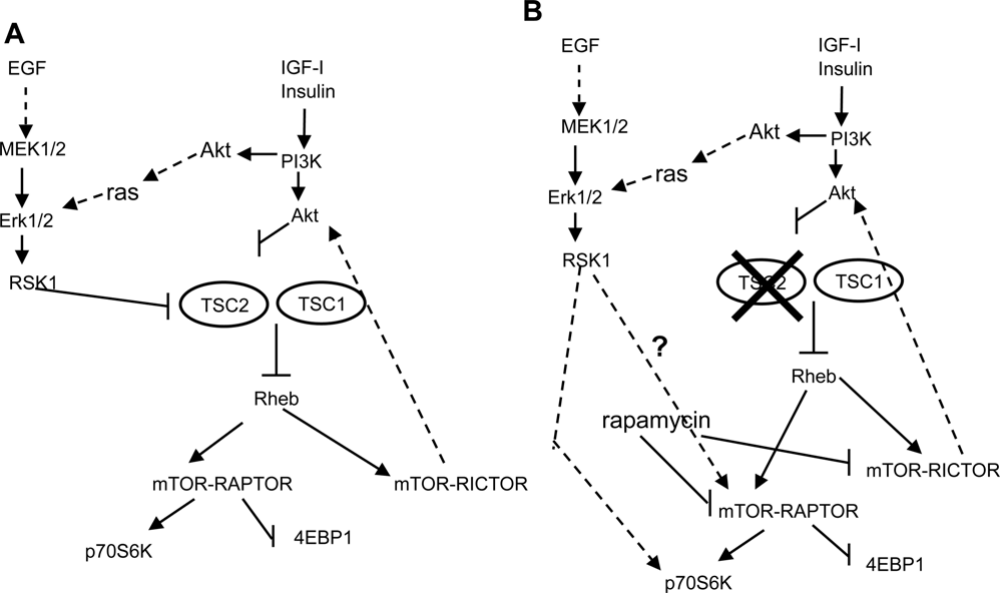
ERK- and PI3K-Akt-dependent cascades regulating mTOR signalling. A: In wild type cells, activation of either pathways result in the phosphorylation of both RSK1 and Akt that, in turn, are capable of phosphorylating tuberin directly. B: In TSC2^−/−^ ASM cells, the loss of tuberin may lead to modification of ERK-RSK1 function with direct/indirect regulation of mTOR and p706K. ERK-RSK1 pathway appears to have a more relevant role while the PI3K cascade, may not be properly regulated. This might explain the EGF requirement for proliferation of TSC2^−/−^ ASM cells.

Serum-stimulated phosphorylation of tuberin was observed in embryonic fibroblasts from Akt1/Akt2 double knockout mice, consistent with the hypothesis that another kinase, such as RSK1, phosphorylates tuberin in response to stimuli that activate the Ras/MAPK pathway [35]. Tuberin is a phosphorylation target of Ras-ERK signalling and phosphorylation results in the suppression of its biochemical and biological tumor-suppressive functions [13]. Viral-driven tuberin expression in TSC2^−/−^ASM cells decreased phosphorylation of p42/44 MAPK. This effect was also observed with anti-EGFR antibody, whereas rapamycin had a similar effect only when added at plating time. The data are consistent with a regulatory mechanism by which the Ras/MAPK and PI3K pathways converge on tuberin to inhibit its function [12]. In human TSC2^−/−^ ASM cells, the lack of tuberin seems to shift the regulation of cell proliferation to the Ras/MAPK pathway while PI3K cascade appears to activate an independent and different cell signalling pathway (Figure 8). This may explain why IGF-I which has a proliferative action in normal smooth muscle cells, fails to promote proliferation in TSC2^−/−^ ASM cells, and activates survival pathways [16]. From our results it may be also inferred a possible modulation of mTOR by the EGF pathway. We may speculate that, in TSC2^−/−^ ASM cells, Akt is highly phosphorylated because its major substrate, tuberin, is absent while the phosphorilative events are particularly active, expecially mTOR-rictor. In this situation Akt is insensitive to PI3K inhibitors but following *TSC2* transfection the sensitivity is restored.

In conclusion, re-expression of tuberin caused a more efficient reversion of phenotypic, proliferative and biochemical characteristics of TSC2^−/−^ASM cells compared to the pharmacological approach. EGF-dependent TSC2^−^/^−^ ASM proliferation is related to the lack of tuberin. The anti-EGFR antibody was more efficient than rapamycin in reducing reactivity to HMB45, the proliferation rate, and in controlling ERK activation in human TSC2^−/−^ASM cells. Both rapamycin and anti-EGFR were equally affected in inhibiting S6 activation, while ERK phosphorylation was more sensitive to anti-EGFR. In TSC2^−^/^−^ ASM cells, the EGF pathway, involving ERK, may play a more relevant role than a PI3K cascade in cell growth.

## Supporting Information

Figure S1 Effect of rapamycin (rapa) on TSC2^−/−^ASM cell growth. Rapamycin addition at the concentrations of 5 ng/ml 3 hours after plating did not have any significant effect on cell growth while 10, and 20 ng/ml slowed TSC2^−/−^ASM proliferation only after 7 days of incubation. Mean values±SEM. Significant differences (*P<0.05) versus control were evaluated by student's t-test
(5.76 MB TIF)

Figure S2 Densitometric analysis of phosphorylation of Akt in TSC2^−/−^ASM cells in complete medium or following 24 hours starvation. Incubation of TSC2^−/−^ASM cells and following TSC2-transfection for 2 hours with IGF-I (50 ng/ml) induced Akt phosphorylation. Incubation with PI3K inhibitor, LY294002 (LY; 20 µM), or ERK inhibitor, PD98059 (PD; 30 µM), did not influence the activation of Akt. Expression of Akt was not altered by any treatment. Mean values±SEM. Significant differences (***P<0.001) versus control were evaluated by student's t-test.
(7.38 MB TIF)

## References

[1] Young J, Povey S (1998). The genetic basis of tuberous sclerosis.. Mol Med Today.

[2] Janssen LAJ, Sampson JR, van der Est M, Deelen W, Verhoef S (1994). Refined localization of TSC1 by combined analysis of 9q34 and 16p13 data in 14 tuberous sclerosis families.. Hum Genet.

[3] European Chromosome 16 Tuberous Sclerosis Consortium (1993). Identification and characterization of the tuberous sclerosis gene on chromosome 16.. Cell.

[4] Gomez MR (1999). Tuberous Sclerosis, 3^rd^ Ed.

[5] Kenerson HL, Aicher LD, True LD, Yeung RS (2002). Activated mammalian target of rapamycin pathway in the pathogenesis of tuberous sclerosis complex renal tumors.. Cancer Res.

[6] van Slegtenhorst M, Nellist M, Nagelkerken B, Cheadle J, Snell R (1998). Interaction between hamartin and tuberin, the TSC1 and TSC2 gene products.. Hum Mol Genet.

[7] Goncharova EA, Goncharov DA, Eszterhas A, Hunter DS, Glassberg MK (2002). Tuberin regulates p70 S6 kinase activation and ribosomal protein S6 phosphorylation. A role for the Tsc2 tumor suppressor gene in pulmonary lymphangeioleiomyomatosis (LAM).. J Biol Chem.

[8] Kwiatkowski DJ, Zhang H, Bandura JL, Heiberger KM, Glogauer M (2002). El-Hashemite, and Onda H. A mouse model of TSC1 reveals sex-dependent lethality from liver hemangiomas, and up-regulation of p70S6 kinase activity in Tsc1 null cells.. Hum Mol Genet.

[9] Gingras AC, Raught B, Sonenberg N (1999). eIF4 initiation factors: effectors of mRNA recruitment to ribosomes and regulators of translation.. Annu Rev Biochem.

[10] Takata M, Ogawa W, Kitamura T, Hino Y, Kuroda S (1999). Requirement for Akt (protein kinase B) in insulin-induced activation of glycogen synthase and phosphorylation of 4E-BP1 (PHAS-1).. J Biol Chem.

[11] Karbowniczek M, Cash T, Cheung M, Robertson GP, Astrinidis A (2004). Regulation of B-Raf kinase activity by tuberin and Rheb is mammalian target of rapamycin (mTOR)-independent.. J Biol Chem.

[12] Roux PP, Ballif BA, Anjum R, Gygi SP, Blenis J (2004). Tumor-promoting phorbol esters and activated Ras inactivate the tuberous sclerosis tumor suppressor complex via p90 ribosomal S6 kinase.. PNAS.

[13] Ma L, Chen Z, Erdjument-Bromage H, Tempst P, Pandolfi PP (2005). Phosphorylation and functional inactivation of TSC2 by Erk: implications for tuberous sclerosis and cancer pathogenesis.. Cell.

[14] Han S, Santos TM, Puga A, Roy J, Thiele EA (2004). Phosphorylation of tuberin as a novel mechanism for somatic inactivation of the tuberous sclerosis complex proteins in brain lesions.. Cancer Res.

[15] Lesma E, Grande V, Carelli S, Brancaccio D (2005). Isolation and growth of smooth muscle-like cells derived from tuberous sclerosis complex-2 human renal angiomyolipoma: EGF is the required growth factor.. Am J Pathol.

[16] Carelli S, Lesma E, Paratore S, Grande V, Zadra G (2007). Survivin expression in tuberous sclerosis complex cells.. Mol Med.

[17] Arbiser JL, Yeung R, Weiss SH, Arbiser ZK, Amin MB (2001). The generation and characterization of a cell line derived from a sporadic renal angiomyolipoma.. Am J Pathol.

[18] Astrinidis A, Cash TP, Hunter DS, Walker CL, Chernoff J (2002). Tuberin, the tuberous sclerosis complex 2 tumor suppressor gene product, regulates Rho activation, cell adhesion and migration.. Oncogene.

[19] Bagby SP, Kirk EA, Mitchell LH, O'Reailly MM, Holden WE (1993). Proliferative synergy of ANG II and EGF in porcine aortic vascular smooth cells.. Am J Physiol.

[20] Inoki K, Li Y, Zhu T, Wu J, Guan KL (2002). TSC2 is phosphorylated and inhibited by Akt and suppresses mTOR signalling.. Nat Cell Biol.

[21] Karbowniczek M, Yu J, Henske EP (2003). Renal angiomyolipomas from patients with sporadic lymphangiomyomatosis contain both neoplatic and non-neoplastic vascular structures.. Am J Pathol.

[22] El-Hashemite N, Walker V, Kwiatkowski DJ (2005). Estrogen enhances whereas tamoxifen retards development of Tsc mouse liver hemangioma: a tumor related to renal angiomyolipoma and pulmonary lymphangioleiomyomatosis.. Cancer Res.

[23] Kuemmerle JF, Bushman TL (1998). IGF-I stimulates intestinal muscle cell growth by activating distinct PI 3-kinase and MAP kinase pathways.. Am J Gastrointest Liver Physiol.

[24] Powis G, Bonjouklian R, Berggren MM, Gallegos A, Abraham R (1994). Wortmannin, a potent and selective inhibitor of phosphatidylinositol-3-kinase.. Canc Res.

[25] Cross MJ, Stewart A, Hodgkin MN, Kerr DJ, Wakelam MJO (1995). Wortmannin and its structural analogue demethoxyviridin inhibit stimulated phospholipase A_2_ activity in Swiss 3T3 cells.. J Biol Chem.

[26] Perugini RA, McDade TP, Vittimberga FJ, Callery MP (2000). Pancreatic cancer cell proliferation is phosphatidylinositol 3-kinase dependent.. J Surg Res.

[27] Stambolic V, Suzuki A, de la Pompa JL, Brothrs GM, Mirtsos C (1998). Negative regulation of PKB/Akt-dependent cell survival by the tumor suppressor PTEN.. Cell.

[28] Zhang H, Cicchetti G, Onda H, Koon HB, Asrican K (2003). Loss of Tsc1/Tsc2 activates mTOR and disrupts PI3K-Akt signaling through downregulation of PDGFR.. J Clin Invest.

[29] Kuemmerle JF, Zhou H, Bowers JG (2004). IGF-I stimulates human intestinal smooth muscle cell growth by regulation of G1 phase cell cycle proteins.. Am J Gastrointest Liver Physiol.

[30] Vokes EE, Chu E (2006). Anti-EGFR therapies: clinical experience in colorectal, lung, and head and neck cancers.. Oncology.

[31] Harwood FC, Shu L, Houghton PJ (2008). mTORC1 signaling can regulate growth factor activation of p44/42 mitogen activated protein kinases through protein phosphatase 2A.. J Biol Chem.

[32] Kenerson HL, Dundon TA, Yeung RS (2005). Effects of rapamycin in the Eker rat model of tuberous sclerosis complex.. Pediatric Res.

[33] Lee L, Sudentas P, Donohue B, Asrican K, Worku A (2005). Efficacy of a rapamycin analog (CCI-779) and IFN-gamma in tuberous sclerosis mouse models.. Genes Chromosomes Cancer.

[34] Bissler J, McCormack FX, Young LR, Elwing JM, Chuck G (2008). Sirolimus for Angiomyolipoma in Tuberous Sclerosis Complex or Lymphangioleiomyomatosis.. N Engl J Med.

[35] Peng XD, Xu PZ, Chen ML, Hahn-Windgassen A, Skeen J (2003). Dwarfism, impaired skin development, skeletal muscle atrophy, delayed bone development, and impeded adipogenesis in mice lacking Akt1 and Akt2.. Genes Dev.

